# *Sarcoptes scabiei* Mites Modulate Gene Expression in Human Skin Equivalents

**DOI:** 10.1371/journal.pone.0071143

**Published:** 2013-08-05

**Authors:** Marjorie S. Morgan, Larry G. Arlian, Michael P. Markey

**Affiliations:** 1 Department of Biological Sciences, Wright State University, Dayton, Ohio, United States of America; 2 Department of Biochemistry and Molecular Biology, Wright State University, Dayton, Ohio, United States of America; Indian Institute of Science, India

## Abstract

The ectoparasitic mite, *Sarcoptes scabiei* that burrows in the epidermis of mammalian skin has a long co-evolution with its hosts. Phenotypic studies show that the mites have the ability to modulate cytokine secretion and expression of cell adhesion molecules in cells of the skin and other cells of the innate and adaptive immune systems that may assist the mites to survive in the skin. The purpose of this study was to identify genes in keratinocytes and fibroblasts in human skin equivalents (HSEs) that changed expression in response to the burrowing of live scabies mites. Overall, of the more than 25,800 genes measured, 189 genes were up-regulated >2-fold in response to scabies mite burrowing while 152 genes were down-regulated to the same degree. HSEs differentially expressed large numbers of genes that were related to host protective responses including those involved in immune response, defense response, cytokine activity, taxis, response to other organisms, and cell adhesion. Genes for the expression of interleukin-1α (IL-1α) precursor, IL-1β, granulocyte/macrophage-colony stimulating factor (GM-CSF) precursor, and G-CSF precursor were up-regulated 2.8- to 7.4-fold, paralleling cytokine secretion profiles. A large number of genes involved in epithelium development and keratinization were also differentially expressed in response to live scabies mites. Thus, these skin cells are directly responding as expected in an inflammatory response to products of the mites and the disruption of the skin’s protective barrier caused by burrowing. This suggests that *in vivo* the interplay among these skin cells and other cell types, including Langerhans cells, dendritic cells, lymphocytes and endothelial cells, is responsible for depressing the host’s protective response allowing these mites to survive in the skin.

## Introduction

The scabies mite, *Sarcoptes scabiei* is a medically and economically important ectoparasite of humans and other mammals worldwide. The mites live in the lower stratum corneum of the skin. All active life stages of the mite (larvae, protonymph, tritonymph and adults) are obligate permanent parasites that require host extracellular fluid (plasma) that seeps into the burrow as nutrition to live [1].

The parasite has had a long co-evolution with its mammalian hosts and has adapted multiple mechanisms for evading the host innate and adaptive immune responses. Hosts exhibit delayed inflammatory and adaptive immune responses to this parasite. In humans, clinical symptoms (skin inflammation) may not appear for 4 to 8 weeks after initial infestation. It is now clear that this delay is the result of the mite’s ability to modulate multiple aspects of the host’s inflammatory and immune responses. Some of these have been elucidated as discussed below.

Cells of the epidermis such as keratinocytes, dendritic cells and Langerhans cells (LCs) are the first cells to encounter the mite and its products. The innate inflammatory and subsequent adaptive responses of the host skin provide the first line of defense against the invasion, survival and reproduction of the mite in the skin. The mites stimulate epidermal keratinocytes and dendritic cells with molecules in their eggs, feces, excreta, saliva and other secretory products (molting enzymes and hormones), the physical activity of the chelicerae, pedipalps and legs as they burrow, and decomposing bodies after they die. However, the live mites have been observed to produce copious amounts of saliva as they burrow and this material is likely the source of immune/inflammation modulating molecules [2]. As products from the mites penetrate the dermis, they stimulate other cells including fibroblasts, endothelial cells of the microvasculature and immune effector cells including LCs, macrophages, mast cells, and lymphocytes [3]–[9]. Presumably, LCs and other dendritic cells take up and process antigens from the mites in the skin and transport them to regional lymphatic tissue where the adaptive immune response is initiated via activation of B- and T-lymphocytes.

Live scabies mites induce secretion of the anti-inflammatory cytokine interleukin-1 receptor antagonist (IL-1ra) from the cells in human skin equivalents (fibroblasts and keratinocytes) [10]. IL-1ra inhibits the activity of the proinflammatory cytokine IL-1 by binding to the IL-1 receptor that is found on many cells including T-cells, B-cells, natural killer cells, macrophages and neutrophils [10], [11].

Scabies mite extract contains molecules that suppress the expression of intercellular and vascular cell adhesion molecules (ICAM-1 and VCAM-1) and E-selectin by cultured normal human endothelial cells of the skin microvasculature [12]. This suppression would inhibit or reduce extravasation of lymphocytes, neutrophils and other cells into the dermis in the vicinity of the mite and thus hinder the host’s ability to mount a successful protective response.

Scabies mites may inhibit co-stimulatory interactions between antigen presenting cells and T-cells. Scabies mite extract induces human T-regulatory cells to produce IL-10 [13]. IL-10 acts as a potent anti-inflammatory cytokine by suppressing the secretion of proinflammatory cytokines and expression of MHC-II molecules on antigen-presenting cells. Thus, the interaction of the MHC-II-antigen complex and the T-cell receptor necessary for activation and proliferation of B-cells into antibody secreting plasma cells would be inhibited/reduced.

Spleen cells from scabies-challenged mice and mice vaccinated with scabies extract exhibited reduced gene expression for B7-2 (CD86) on B-cells and its receptor, CD28 on T-cells [14]. In addition, genes for expression of CD40 on B-cells and its receptor, CD40L on T-cells, were down-regulated. These co-signals accompany the T-cell receptor MHC-II-antigen complex coupling that activates a B-cell to become an antibody-producing plasma cell.

Human skin equivalents and monocultures of normal human epidermal keratinocytes and dermal fibroblasts increase secretion of vascular endothelial growth factor (VEGF) in response to live scabies mites and mite extracts [10], [15]. VEGF would increase vascularity and fluid (plasma) in the mite burrow in the vicinity of the mouthparts and oral opening of the mite. We believe this fluid is the main source of water and nutrition for the mite in the otherwise dry stratum corneum [1]. Evidence for this is that the mites ingest antibody from the host presumably in this plasma [16].

Scabies mite products may be able to reduce IL-8 activity in the vicinity of the scabietic lesion in two ways. IL-8 is a chemokine that is chemotactic for the extravasation of neutrophils to the site of a pathogen. Monocultures of human epidermal keratinocytes, dermal fibroblasts, microvascular endothelial cells of the skin, and dendritic cells challenged with scabies extract exhibited reduced levels of IL-8 in the media when compared to unchallenged controls [12], [15], [17], [18]. Also, there is evidence that these mites may produce an IL-8 binding protein that may reduce local IL-8 levels thereby inhibiting chemotaxis of neutrophils (unpublished).

Scabies gut serine protease inhibitors of the serpin superfamily have been found to bind to several plasma proteins in the complement cascades and block the three pathways (classical, alternative and lectin) of the human complement system [19]. Because scabies mites ingest plasma [16], inactivating host complement may protect the mite gut from complement-mediated damage. Complement inhibition may promote the pyoderma caused by group A streptococci that is often associated with scabies lesions [20].

As is evident, many aspects of the phenotypic response to these mites have been elucidated by studies of the responses of cultured keratinocytes, fibroblasts, endothelial cells, lymphocytes, monocytes and dendritic cells derived from them as well as studies of gene expression of spleen cells and cytokine profiles of lymph node cells by flow cytometry. What is clear from these studies is that mite products modulate many aspects of the host protective response. Some of these responses have been identified but there are likely many more.

In contrast to having the ability to down-regulate some aspects of the host’s protective response, extract and live mites themselves can up-regulate secretion of proinflammatory cytokines from keratinocytes, fibroblasts and endothelial cells. Thus, the actual host response seems to be a balance between events that both induce and inhibit the protective response and there seem to be many targets for modulation by molecules from the mite. The duration of the infestation and mite burden play a role in shifting this balance. There is a network of pathways involved in the host innate and adaptive immune responses to mite antigens. Scabies antigens and molecules that modulate the host response appear to influence many points in the networks.

Human skin equivalents (HSEs) consist of an epidermis of stratum corneum and proliferating keratinocytes grown above a dermis of fibroblasts in a collagen matrix and have the properties of human skin. Our previous report [10] was limited in that only the secretion of selected cytokines by HSEs was monitored. In order to facilitate identification of other possible innate phenotypic responses, in the present study we investigated the gene expression in response to live scabies mites in the same HSEs whose cytokine secretion was previously reported [10]. This allowed us to elucidate many more aspects of the inflammatory immune response to burrowing scabies mites in order to develop a broader picture of the mites’ immune and inflammation modulating ability. These data will also identify additional specific sites that may have potential for developing vaccines for the prevention and treatment of this disease.

## Materials and Methods

### Scabies Mites and Extract

Live *Sarcoptes scabiei* variety *canis* mites were collected by aspiration onto a 38 µm (400 mesh) stainless steel screen after they had migrated from skin crusts collected from infested rabbits. For inoculation onto HSEs, the live mites collected onto the screen were washed by aspiration of sequential 4 mL volumes of phosphate-buffered saline with 0.05% Tween-20, endotoxin-free water (Lonza, Walkersville, MD) and 70% ethanol. For extract preparation, mites were washed as above, killed by freezing and later were ground on ice in a Ten Broeck homogenizer in 20 volumes (W/V) endotoxin-free water as previously described [21]. The soluble material was collected following centrifugation and the supernatant (extract) was sterile filtered into sterile vials. Protein concentration of the extract was determined by the Bradford protein assay using bovine serum albumin as the standard [22].

### HSE Challenge

The HSEs used in this study are the same as those whose cytokine secretion profiles were previously reported [10]. EpiDerm EFT-400 full-thickness HSEs and medium were purchased from MatTek (Ashland, MA). Upon arrival, HSEs on their supports were transferred to new 6-well culture plates containing 3.0 mL of fresh medium per well and plates were placed in a 37°C incubator with 5–7% CO_2_. The next day, HSEs were transferred to new plates containing 5.0 mL of fresh medium. At the beginning of the experiment, one set of HSEs (n = 5) were inoculated on their surfaces with several hundred live scabies mites that had been washed and surface-sterilized with 70% ethanol. A second set (n = 5) was inoculated on the surface with 100 µg of soluble scabies extract protein. A third set (n = 4) remained uninoculated and served as controls. HSEs were maintained for 48 h at 37°C with 5–7% CO_2_. At 6, 12, 18, and 24 hours after inoculation, each HSE on its support was lifted from the well, the medium was mixed and a 550 µL aliquot was removed from the sample well and frozen at −80°C for subsequent cytokine secretion measurement as previously reported [10]. At the conclusion of the challenge, the HSE was lifted from its support and cut into pieces which were submerged in 1.5 mL of RNA*later* solution (Ambion Life Technologies, Grand Island, NY). Samples were stored at −80°C until processed for gene expression analysis.

### Gene Expression Analysis

Four HSE replicates for the uninfested control condition and five HSE replicates for each of the test conditions (live mites, mite extract) were processed for gene expression analysis. Each individual HSE was homogenized in 1 mL Trizol (Life Technologies) with the aid of a rotor-stator homogenizer. Following the manufacturer’s protocol, the aqueous phase from Trizol purification was transferred to EZNA RNA purification columns (Omega Bio-Tek, Norcross, GA) and purified using the manufacturer’s protocol. Purified RNA was DNase treated to remove any contaminating genomic DNA using Turbo DNA-Free (Life Technologies). RNA was quantified by spectrophotometry and on an Agilent 2100 Bioanalyzer (Agilent Technologies, Santa Clara, CA). All samples had RIN numbers >8.0.

For each sample, 50 ng was used as input for the Ambion WT Expression Kit (Life Technologies). These were hybridized to the Affymetrix Human Gene 1.0ST arrays (RNA from one HSE sample per gene chip) according to manufacturer’s protocols. Briefly, RNA was reverse transcribed using proprietary semi-random primers to generate a first strand cDNA while adding a T7 promoter and avoiding amplification of rRNA. DNA polymerase was used with this single-strand cDNA to generate double stranded cDNA, and then the RNA was degraded by RNase H. This dscDNA was then *in vitro* transcribed using T7 RNA polymerase to generate cRNA. After a bead-based purification (still using the Ambion WT Expression Kit) and performing the recommended bioanalyzer quality control (Agilent RNA Nano chip) for cRNA size, the antisense cRNA was randomly primed to generate “2nd-cycle” cDNA. The RNA was destroyed by RNase H, and the 2nd cycle cDNA was purified, again using the Ambion protocol. After another check of yield and size distribution by bioanalyzer, the 2nd cycle cDNA was labeled, fragmented, and hybridized to Affymetrix Human Gene 1.0ST GeneChips using the Affymetrix GeneChip Whole Transcript Sense Target Labeling Assay protocol (Affymetrix, PN 701880) for the 169-format GeneChips. All wash and stain reagents were also obtained directly from Affymetrix and used according to this manufacturer’s protocols and the recommended fluidics protocols (FS450_0007 on an Affymetrix Fluidics Station 450). GeneChips were scanned immediately after fluidics on an Affymetrix GeneChip 3000 7G scanner. Data from these arrays has been deposited at the Gene Expression Omnibus (GEO, https://www.ncbi.nlm.nih.gov/geo) and made publically available. The accession number is GSE48459.

Data analysis was carried out in AltAnalyze version 2.0 [23]. This processes raw CEL files from the scanned arrays using the RMA algorithm [24]–[26]. Probesets with DABG (detection above background) p-values above 0.5 or non-log expression below 1 were removed from the analysis. Gene expression levels were determined using constitutive probesets (to summarize exon-level data from the arrays at the gene-level). Gene annotation was derived from the current version of Ensembl [27] or release 54 in a handful of cases where the Affymetrix probe set does not correlate to a current identifier (these were pseudogenes in each case). Replicates were assigned to groups and pairwise comparisons were performed for live mites vs. controls and extract vs. controls.

Beginning with the lists of genes significantly differentially regulated between test and control groups (as determined in AltAnalyze above), gene functions that appeared significantly more often were determined by gene ontology (GO) over representation analysis (ORA), performed using GO-Elite v.1 Beta [28]. Maximum raw p-value was set at 0.05. Minimum fold change was 2.0. GO terms and pathway rankings were pruned by z-score with a cutoff of 1.96. Minimum number of changed genes in a term was set at 3. Two-thousand (2000) ORA permutations were performed, and the primary relational gene database was Ensembl.

## Results

### Live Mites on the HSE Surface

**Figure 1:**
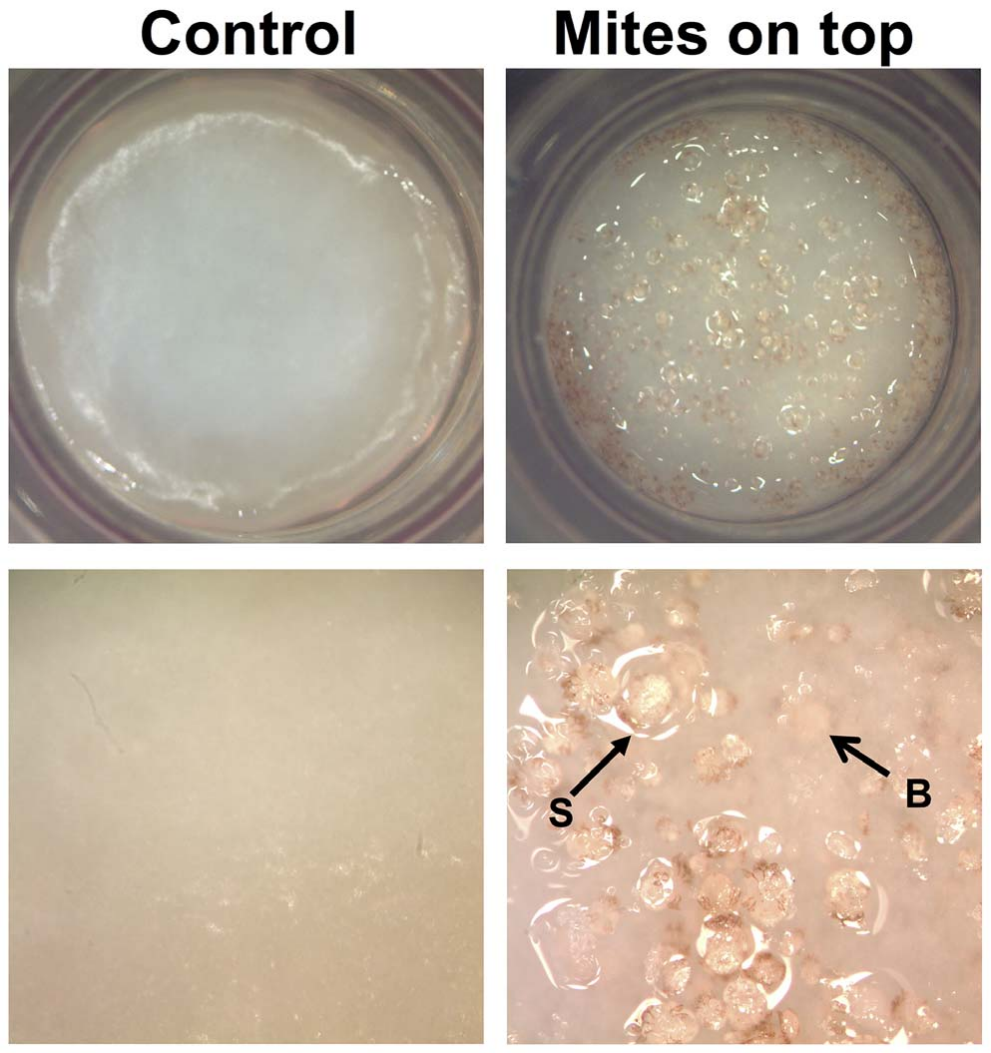
Human skin equivalents (HSEs). Uninoculated controls (left panels) and HSEs inoculated with mites on the top surface (right panels). Mites on the surface (S) or burrowed into (B) the HSE are indicated with arrows.

In this study, live mites placed on the surface of the HSE burrowed into the epidermis of the skin equivalent (Fig. 1) and induced changes in gene expression that were measured at 48 h post-inoculation. Overall, of the more than 25,800 genes measured, 189 genes were up-regulated >2-fold (Table 1) in response to scabies mite burrowing while 152 genes were down-regulated to the same degree (Table 2).

**Table 1:** Genes most up-regulated at 48 h by human skin equivalents in response to live scabies mites (all >2-fold vs. uninoculated controls). Top 50 and selected others of the 189>2x controls.

Rank	Gene Symbol	Definition	Fold×Cont
**1**	KRT37	Keratin, type I cuticular Ha7	8.46
**2**	IL1F9	Interleukin-1 family member 9 (IL-1F9)	8.06
**3**	IL1B	Interleukin-1 beta Precursor (IL-1β)	7.42
**4**	PRSS22	Brain-specific serine protease 4 Precursor (BSSP-4)	7.39
**5**	ARG1	Arginase-1 (Type I arginase)	7.21
**6**	AC011443.6	Iron/zinc purple acid phosphatase-like protein Precursor	6.10
**7**	CLDN17	Claudin-17	5.08
**8**	AL133512.10		4.90
**9**	LCE3C	Late cornified envelope protein 3C	4.82
**10**	HBEGF	Proheparin-binding EGF-like growth factor Precursor	4.71
**11**	SLC7A2	Low affinity cationic amino acid transporter 2 (CAT-2)	4.60
**12**	AC006032.2		4.03
**13**	FCHSD1	FCH and double SH3 domains protein 1	4.02
**14**	RASGEF1B	Ras-GEF domain-containing family member 1B (GPI γ-4)	3.99
**15**	CSF2	Granulocyte-macrophage colony-stimulating factor Precursor (GM-CSF)	3.99
**16**	AGPAT9	1-acyl-sn-glycerol-3-phosphate acyltransferase theta	3.96
**17**	IL1RL1	Interleukin-1 receptor-like 1 Precursor	3.75
**18**	LCE3A	Late cornified envelope protein 3A (Late envelope protein 13)	3.71
**19**	AC026689.6		3.67
**20**	AC004510.1	Urothelial cancer associated 1 (UCA1)	3.63
**21**	KIAA1239	Leucine-rich repeat and WD repeat-containing protein KIAA1239	3.62
**22**	RHCG	Ammonium transporter Rh type C	3.54
**23**	SELE	E-selectin Precursor (Endothelial leukocyte adhesion molecule 1; ELAM-1)	3.54
**24**	RNF39	RING finger protein 39 (Protein HZFw)	3.53
**25**	GPR160	Probable G-protein coupled receptor 160	3.46
**26**	TNIP3	TNFAIP3-interacting protein 3 (A20-binding inhibitor of NF-kappa-B activation 3)	3.37
**27**	RNASE13	Ribonuclease-like protein 13 Precursor	3.24
**28**	IL1A	Interleukin-1 alpha Precursor (IL-1α)	3.24
**29**	APOBEC3A	Probable DNA dC->dU-editing enzyme APOBEC-3A (Phorbolin-1)	3.23
**30**	PLA2G4D	Cytosolic phospholipase A2 delta (cPLA2-δ)	3.20
**31**	TRPV3	Transient receptor potential cation channel subfamily V member 3 (TrpV3)	3.17
**32**	PDCD5	Programmed cell death protein 5 (TF-1 cell apoptosis-related protein 19)	3.16
**33**	DHRS9	Dehydrogenase/reductase SDR family member 9 Precursor	3.13
**34**	ARRDC4	Arrestin domain-containing protein 4	3.11
**35**	OLAH	S-acyl fatty acid synthase thioesterase, medium chain	3.11
**36**	CCL20	C-C motif chemokine 20 Precursor (MIP-3α)[Contains CCL20(1-67)]	3.09
**37**	KLK9	Kallikrein-9 Precursor (Kallikrein-like protein; (KLK-L3)	3.06
**38**	HEPHL1	Hephaestin-like protein 1 Precursor	3.06
**39**	OASL	59 kDa 2′-5′-oligoadenylate synthetase-like protein (TRIP-14)	3.04
**40**	U1	U1 spliceosomal RNA	3.01
**41**	LIPK	Lipase member K Precursor	2.98
**42**	VNN3	Vascular non-inflammatory molecule 3 Precursor (Vanin-3)	2.97
**43**	CXCL5	C-X-C motif chemokine 5 Precursor (Epithelial-derived neutrophil-activating protein 78)	2.93
**44**	LIF	Leukemia inhibitory factor Precursor (LIF)	2.92
**45**	CHRNB1	Acetylcholine receptor subunit beta Precursor	2.91
**46**	ZNF208	Zinc finger protein 208	2.89
**47**	DAZL	Deleted in azoospermia-like (DAZ-like autosomal)	2.87
**48**	ESM1	Endothelial cell-specific molecule 1 Precursor (ESM-1 secretory protein)	2.85
**49**	ELF3	ETS-related transcription factor Elf-3 (ESE-1)	2.83
**50**	IL23A	Interleukin-23 subunit alpha Precursor (IL-23 subunit α)	2.83
**52**	CSF3	Granulocyte colony-stimulating factor Precursor (G-CSF)	2.80
**69**	CCL5	C-C motif chemokine 5 Precursor (T-cell-specific protein RANTES)	2.63
**79**	IL11	Interleukin-11 Precursor (IL-11)	2.46
**84**	IL13RA2	Interleukin-13 receptor alpha-2 chain Precursor (Interleukin-13-binding protein)	2.43
**85**	TGFB2	Transforming growth factor beta-2 Precursor (TGF-β2)	2.43
**86**	CXCL6	C-X-C motif chemokine 6 Precursor (Granulocyte chemotactic protein 2; GCP-2)	2.42
**96**	IL20	Interleukin-20 Precursor (IL-20)	2.37
**124**	CCL3	C-C motif chemokine 3 Precursor (Macrophage inflammatory protein 1-alpha; MIP-1α)	2.19
**150**	LCE2D	Late cornified envelope protein 2D (Late envelope protein 12)	2.12
**156**	KRT75	Keratin, type II cytoskeletal 75	2.11
**160**	CXCL2	C-X-C motif chemokine 2 Precursor (Macrophage inflammatory protein 2-alpha; MIP-2α)	2.10
**163**	KRT38	Keratin, type I cuticular Ha8 (Hair keratin, type I Ha8)	2.09
**184**	LCE3D	Late cornified envelope protein 3D (Late envelope protein 16)	2.01
**186**	IL1F6	Interleukin-1 family member 6 (IL-1F6)	2.01

**Table 2:** Genes most down-regulated at 48 h by human skin equivalents in response to live scabies mites (all >2-fold vs. uninoculated controls). Top 50 and selected others of the 152>2× controls.

Rank	Gene Symbol	Definition	Fold×Cont
**1**	CYP3A7	Cytochrome P450 3A7	−6.03
**2**	AKR1B10	Aldo-keto reductase family 1 member B10	−5.03
**3**	LAMB4	Laminin subunit beta-4 Precursor (Laminin beta-1-related protein)	−5.00
**4**	DAPL1	Death-associated protein-like 1 (Early epithelial differentiation-associated protein)	−4.67
**5**	AADAC	Arylacetamide deacetylase (AADAC)	−4.29
**6**	HTR3A	5-hydroxytryptamine receptor 3A Precursor (5-HT3R)	−4.22
**7**	C10orf99	Putative uncharacterized protein C10orf99 Precursor	−4.01
**8**	CXCL12	Stromal cell-derived factor 1 Precursor (SDF-1)(C-X-C motif chemokine 12)	−3.93
**9**	ANKRD18A	Ankyrin repeat domain-containing protein 18A	−3.76
**10**	AC108671.5	Putative nmrA-like family domain-containing protein	−3.69
**11**	AL359075.15	Putative uncharacterized protein ENSP00000383760 Fragment	−3.51
**12**	ALDH3A1	Aldehyde dehydrogenase, dimeric NADP-preferring (ALDHIII)	−3.29
**13**	PARP9	Poly [ADP-ribose] polymerase 9 (PARP-9)	−3.15
**14**	40790	Septin-4 (Cell division control-related protein 2)	−3.09
**15**	ANK2	Ankyrin-2 (Brain ankyrin; Ankyrin-B; Non-erythroid ankyrin)	−3.08
**16**	GATM	Glycine amidinotransferase, mitochondrial Precursor (Transamidinase)	−3.06
**17**	PXMP4	Peroxisomal membrane protein 4 (24 kDa peroxisomal intrinsic membrane protein)	−3.05
**18**	GBP6	Guanylate-binding protein 6 (GTP-binding protein 6)	−3.01
**19**	AC105921.5-6		−2.88
**20**	CA13	Carbonic anhydrase 13	−2.85
**21**	KRT77	Keratin, type II cytoskeletal 1b (Cytokeratin-1B; Keratin-77)	−2.82
**22**	SEPP1	Selenoprotein P Precursor (SeP)	−2.80
**23**	GNAL	Guanine nucleotide-binding protein G subunit alpha, olfactory type	−2.77
**24**	SLC44A3	Choline transporter-like protein 3	−2.77
**25**	MX1	Interferon-induced GTP-binding protein Mx1	−2.68
**26**	ANK3	Ankyrin-3 (ANK-3; Ankyrin-G)	−2.68
**27**	U4	U4 spliceosomal RNA	−2.67
**28**	APOB	Apolipoprotein B-100 Precursor (Apo B-100)	−2.67
**29**	THEM5	Thioesterase superfamily member 5	−2.67
**30**	PSAT1	Phosphoserine aminotransferase	−2.64
**31**	CYP2C18	Cytochrome P450 2C18	−2.64
**32**	CYP3A4	Cytochrome P450 3A4	−2.62
**33**	IFIT1	Interferon-induced protein with tetratricopeptide repeats 1 (IFIT-1)	−2.61
**34**	AC013251.19-1		−2.59
**35**	PSAPL1	Proactivator polypeptide-like 1 Precursor	−2.58
**36**	AK5	Adenylate kinase isoenzyme 5 (AK 5)	−2.58
**37**	OLFML1	Olfactomedin-like protein 1 Precursor	−2.56
**38**	GSTO2	Glutathione S-transferase omega-2 (GSTO-2)	−2.56
**39**	SP110	Sp110 nuclear body protein (Transcriptional coactivator Sp110)	−2.54
**40**	IDH1	Isocitrate dehydrogenase [NADP] cytoplasmic (IDH)	−2.53
**41**	PYDC1	Pyrin domain-containing protein 1 (Pyrin-only protein 1)	−2.52
**42**	BX005195.5	Novel protein similar to contactin associated protein-like 3 (CNTNAP3) Fragment	−2.52
**43**	AL953854.21	Novel protein similar to contactin associated protein-like 3 (CNTNAP3) Fragment	−2.52
**44**	BRCA1	Breast cancer type 1 susceptibility protein (RING finger protein 53)	−2.51
**45**	CNTN1	Contactin-1 Precursor (Neural cell surface protein F3; Glycoprotein gp135)	−2.51
**46**	PCLO	Protein piccolo (Aczonin)	−2.50
**47**	BBOX1	Gamma-butyrobetaine dioxygenase (γ-butyrobetaine hydroxylase)	−2.49
**48**	ASB5	Ankyrin repeat and SOCS box protein 5 (ASB-5)	−2.48
**49**	ADH1C	Alcohol dehydrogenase 1A (Alcohol dehydrogenase subunit alpha)	−2.42
**50**	Y_RNA	Y RNA	−2.42
**53**	CYP3A5	Cytochrome P450 3A5	−2.41
**56**	KRT15	Keratin, type I cytoskeletal 15 (Cytokeratin-15)	−2.37
**61**	TLR3	Toll-like receptor 3 Precursor (CD283 antigen)	−2.33
**64**	CYP2J2	Cytochrome P450 2J2 (Arachidonic acid epoxygenase)	−2.32
**86**	IL12RB2	Interleukin-12 receptor beta-2 chain Precursor (IL-12 receptor β-2)	−2.20
**88**	KRT13	Keratin, type I cytoskeletal 13 (Cytokeratin-13)	−2.20
**90**	CXCL14	C-X-C motif chemokine 14 Precursor (Chemokine BRAK)	−2.19
**118**	KRT1	Keratin, type II cytoskeletal 1 (Cytokeratin-1)	−2.09
**129**	TNFSF10	Tumor necrosis factor ligand superfamily member 10 (TRAIL)	−2.06
**143**	ANKRD18B	Ankyrin repeat domain-containing protein 18B	−2.02
**148**	IL16	Interleukin-16 Precursor (IL-16)	−2.01

We determined that the genes for expression of IL-1α precursor, IL-1β, GM-CSF precursor, and G-CSF precursor were up-regulated 2.8- to 7.4-fold (Table 1). The IL1F9 gene was expressed 8-fold more than in controls and was the second most up-regulated gene. This gene codes for a protein in the IL-1 cytokine family. Scabies mites induced a 3.75-times up-regulated expression of the gene IL1RL1 (IL-1 receptor-like precursor) above its constitutive expression. Likewise, the genes for the receptors IL1R2 and IL13RA2 were also up-regulated >2-fold (Table 1).

The gene most up-regulated was KRT37 (also know as Ha 7). This is a keratin family gene that codes for the type I keratin protein which heterodimerizes with type II keratin to form hair and nails. The LCE3C gene encoding for late cornified envelope protein 3C was up-regulated 4.8-fold above constitutive and several other LCE genes (LCE3A, LCE2D, LCE3D) were also up-regulated by >2-fold.

Conversely, several genes that encode for keratins and are associated with keratinization were down-regulated (Table 2). The two genes KRT13 and KRT15, which code for type I cytokeratins, were down-regulated by 2.20 and 2.37 times below constitutive expression. Likewise, the gene KRT77 that codes for a type II keratin, type II cytoskeletal 1b protein, was down-regulated 2.82 times as was KRT1 (2.08-fold down). Several members of the Ankyrin (ANK2, ANK3, ASB5, ANKRD18A and ANKRD18B) and Cytochrome P450 (CYP2C18, CYP2J2, CYP3A4, CYP3A5 and CYP3A7) gene families were also down-regulated >2-fold.

Gene ontology (GO) groups provide a useful framework for describing the cellular component (C), biological process (P), and molecular function (F) influenced by the expression of a particular set of genes. The 25 GO groups in which the highest percentage of genes were differentially expressed by skin equivalents parasitized with burrowing scabies mites are shown in Table 3. Greater than 87% of the genes in all GO groups were measured and for 23 of the 25 most highly ranked groups more than 95% were present. Of the top 15 gene groups expressed, 10 included genes involved in the regulation of the secretion of cytokines. The GO group of genes involved in cytokine activity included 23 genes for cytokines that were changed in HSEs that were challenged by live scabies mites. Six groups included genes for expression of IL-1α while 14 groups included genes for expression of IL-1β. Of note was that 11% of genes involved in keratinization and 9% of genes in the taxis GO groups changed expression in response to live scabies mites.

**Table 3:** Gene Ontology (GO) groups in which the highest percentage of genes were differentially expressed by human skin equivalents exposed to live scabies mites.

Rank	GO Type	GO Name	# in GO	# Measured	% Present	# Changed	% Changed	Gene symbols of changed genes
**1**	F	interleukin-1 receptor antagonist activity	9	9	100	3	33.3	IL1B, IL1F6, IL1F9
**2**	P	juvenile hormone secretion	24	24	100	7	29.2	ACVR1C, EDN1, FST, IL11, IL1B, INHBA, LIF
**3**	P	positive regulation of tumor necrosis factor production	13	13	100	3	23.1	CLEC7A, FCER1G, TLR3
**4**	P	negative regulation of secretion	41	38	93	8	21.1	ACVR1C, EDN1, FST, IL11, IL1B, INHBA, LIF, SRGN
**5**	P	positive regulation of interleukin-6 production	17	17	100	3	17.6	FCER1G, IL1B, TLR3
**6**	P	vascular endothelial growth factor production	19	18	95	3	16.7	ACE2, IL1A, IL1B
**7**	F	aromatase activity	25	25	100	4	16.0	CYP2C18, CYP2J2, CYP3A5, CYP3A7
**8**	P	positive regulation of angiogenesis	20	20	100	3	15.0	FGF2, IL1A, IL1B
**9**	P	foam cell differentiation	37	36	97	5	13.9	ABCG1, APOB, CSF2, INHBA, LIF
**10**	P	positive regulation of MAPKKK cascade	39	37	95	5	13.5	IL11, IL1B, LIF, TGFB2, TLR3
**11**	P	regulation of vasoconstriction	24	23	96	3	13.0	ACE2, EDN1, HTR2A
**12**	P	cellular aldehyde metabolic process	23	23	100	3	13.0	AKR1B10, ALDH3A1, IDH1
**13**	P	positive regulation of JAK-STAT cascade	24	24	100	3	12.5	CSF2, IL20, LIF
**14**	F	cytokine activity	208	197	95	23	11.7	BMP2, CCL20, CCL3, CCL5, CSF2, CSF3, CXCL12, CXCL14, CXCL2, CXCL5, CXCL6, IL11, IL16, IL1A, IL1B, IL1F6, IL1F9, IL20, IL23A, INHBA, LIF, TGFB2, TNFRSF11B
**15**	P	regulation of cytokine secretion	29	26	90	3	11.5	IL1A, PYDC1, SRGN
**16**	F	oxygen binding	37	36	97	4	11.1	CYP2C18, CYP3A4, CYP3A5, CYP3A7
**17**	P	keratinization	92	91	99	10	11.0	AHNAK2, BMP2, FGF2, FST, IL20, LCE2D, LCE3A, LCE3C, LCE3D, SPRR2D
**18**	P	positive regulation of nuclear division	32	28	88	3	10.7	EDN1, IL1A, IL1B
**19**	P	platelet activating factor secretion	41	38	93	4	10.5	ACE2, IL1A, PYDC1, SRGN
**20**	P	positive regulation of lipid metabolic process	30	29	97	3	10.3	ABCG1, APOB, IL1B
**21**	P	nitric oxide biosynthetic process	35	31	89	3	9.7	EDN1, IL1B, SLC7A2
**22**	P	positive regulation of protein amino acid phosphorylation	78	76	97	7	9.2	BMP2, CSF2, FGF2, IL11, IL1B, IL20, LIF
**23**	P	negative regulation of transport	108	99	92	9	9.1	ACVR1C, EDN1, FST, HTR2A, IL11, IL1B, INHBA, LIF, SRGN
**24**	P	taxis	213	209	98	18	8.6	AC130360.4-3, CCL20, CCL3, CCL5, CXCL12, CXCL14, CXCL2, CXCL5, CXCL6, DEFB4, FCER1G, FGF2, GPR77, IL16, IL1B, ITGA1, TGFB2, TPM3
**25**	P	group transfer coenzyme metabolic process	35	35	100	3	8.6	GBA2, VNN1, VNN3

GO types are characterized according to the gene product’s involvement in biological processes (P) or molecular functions (F).

The 25 GO groups of genes that had the greatest number of genes that changed expression in response to scabies mites are listed in Table 4. In response to burrowing scabies mites, HSEs differentially expressed large numbers of genes that were related to host protective responses including those involved in immune response, defense response, cytokine activity, taxis, response to other organisms, and cell adhesion. A large number of genes involved in epithelium development and keratinization were also differentially-expressed in response to live scabies mites.

**Table 4:** Gene Ontology (GO) groups in which the greatest number of genes were differentially expressed by human skin equivalents exposed to live scabies mites.

Rank	GO Type	GO Name	# in GO	# Measured	% Present	# Changed	% Changed
**1**	P	immune response	884	771	87	47	6.1
**2**	C	extracellular space	604	576	95	37	6.4
**3**	P	defense response	734	673	92	34	5.1
**4**	P	regulation of body fluid levels	958	939	98	31	3.3
**5**	F	cytokine activity	208	197	95	23	11.7
**6**	P	epithelium development	458	450	98	21	4.7
**7**	P	cellular lipid metabolic process	726	707	97	21	3.0
**8**	P	taxis	213	209	98	18	8.6
**9**	P	response to other organism	273	263	96	18	6.8
**10**	P	oxidation reduction	625	599	96	17	2.8
**11**	P	branching morphogenesis of a tube	303	297	98	16	5.4
**12**	P	cellular amino acid and derivative metabolic process	424	408	96	16	3.9
**13**	P	polyphosphate biosynthetic process	602	593	99	16	2.7
**14**	P	leukocyte activation	372	361	97	15	4.2
**15**	P	regulation of phosphorylation	420	414	99	15	3.6
**16**	C	endoplasmic reticulum membrane	550	537	98	15	2.8
**17**	P	polyphenic determination	584	569	97	15	2.6
**18**	P	developmental growth involved in morphogenesis	248	242	98	14	5.8
**19**	P	regulation of cell cycle	365	359	98	13	3.6
**20**	F	electron carrier activity	287	272	95	12	4.4
**21**	P	pigment accumulation in tissues	185	178	96	11	6.2
**22**	C	microsome	195	188	96	11	5.9
**23**	P	positive regulation of cell proliferation	226	224	99	11	4.9
**24**	P	cell-cell adhesion	342	331	97	11	3.3
**25**	P	keratinization	92	91	99	10	11.0

GO types are characterized according to the gene product’s involvement in cellular components (C), biological processes (P), or molecular functions (F).

### Scabies Mite Extract on the HSE Surface

When compared to uninoculated controls, only 15 genes were differentially expressed by HSEs exposed to soluble scabies mite extract (Table 5). No genes were significantly (>2-fold) down-regulated. Eight of the 15 up-regulated genes were also expressed more by HSEs exposed to burrowing scabies mites including KRT37, IL1F9, ARG1, AC011443.6 and LCE3C. Interestingly, IL1B gene expression was not changed (1.06-fold vs. control).

**Table 5:** Genes up-regulated at 48 h by human skin equivalents in response to scabies mite extract (all >2-fold vs. uninoculated controls).

Rank	Gene Symbol	Definition	Fold×Cont
**1**	ZNF208	Zinc finger protein 208	2.73
**2**	ARMC4	Armadillo repeat-containing protein 4	2.69
**3**	LCE3C	Late cornified envelope protein 3C	2.41
**4**	KRT37	Keratin, type I cuticular Ha7	2.40
**5**	ARG1	Arginase-1 (Type I arginase)	2.38
**6**	GSTT2	Glutathione S-transferase theta 2 (GSTT2)	2.25
**7**	GSTT2B	Glutathione S-transferase theta-2	2.25
**8**	UPK1A	Uroplakin-1a	2.23
**9**	TRAPPC2	Trafficking protein particle complex subunit 2	2.21
**10**	AC011443.6	Iron/zinc purple acid phosphatase-like protein Precursor	2.18
**11**	U6	U6 spliceosomal RNA	2.10
**12**	AC116654.4-4	Immunoglobulin Kappa light chain V gene segment	2.06
**13**	RNF39	RING finger protein 39	2.05
**14**	FAM70A	Protein FAM70A	2.02
**15**	IL1F9	Interleukin-1 family member 9 (IL-1F9)	2.00

## Discussion

Studies using cultured normal human epidermal keratinocytes and dermal fibroblasts and dermal endothelial cells of the microvasculature show a modulation of cytokine secretion and expression of cell adhesion molecules in response to molecules in scabies mite extracts [12], [15], [18], [21]. In those studies, the cytokines and adhesion molecules measured were selected because they were known to play key roles in immune and inflammatory responses. However, as a result of those initial studies, it is now apparent that the possible phenotypic responses to scabies mites and their products are many. It would be a laborious task to select, identify and quantify so many of these. In addition, these previous studies did not determine if mites affect the expression of the receptors for these cytokines. Also, the use of monocultures of cells in a liquid media required the use of extracts made from crushed mite bodies because the mites themselves would not survive in the culture media of the cells. These limitations led us to use a skin model to investigate cytokine secretion [10] and gene expression by skin cells in response to live scabies mites. The use of the HSEs also allows for the cell-cell interaction between epidermal keratinocytes and dermal fibroblasts, as is the case *in vivo.*

Human skin equivalents have physical properties similar to the epidermis and dermis of normal human skin. They consist of an epidermis of stratum corneum above keratinocytes layered over a dermis of fibroblasts. Scabies mites will readily burrow into the epidermis of these HSEs. Thus, the combined inflammatory and innate immune responses of the keratinocytes and fibroblasts and their interactions can be studied in response to burrowing live scabies mites and their products such as saliva and fecal material. Our experiments used one set of HSEs to determine both the cytokine secretion as previously reported in [10] and gene expression responses of normal human epidermal keratinocytes and dermal fibroblasts when these tissues were challenged for 48 h with live mites and their products. By looking at gene expression, many more possible phenotypic responses could then be considered for phenotypic studies. We recognize that expression of a gene may not lead to a measurable phenotypic effect. But, given the long co-evolution of these mites with their mammalian hosts, it provides a bigger picture of the potential affects these mites have on modulating the phenotypic response of the host’s structural cells of the skin and cells involved in inflammatory and immune responses.

This study found that scabies mites influence expression of numerous genes. A particularly interesting finding of this study was the 7.4-fold increase in IL1B gene expression in response to burrowing scabies mites. These same HSEs exhibited a 4-fold increase in the secretion of this cytokine onto their surfaces [10]. The IL1B gene is a member of GO groups involved in the modulation of IL-1ra activity, positive regulation of IL-6 activity and VEGF production (Table 3). These HSEs also secreted increased amounts of IL-1ra, IL-6 and VEGF onto their surfaces in response to burrowing scabies mites [10], perhaps modulated through the increased IL1B gene expression. IL-1β also stimulates the expression of the IL1F9 gene and the secretion of this cytokine by keratinocytes [29] and we observed an 8-fold increase in IL1F9 expression by these HSEs. This cytokine can activate fibroblasts to produce CCL20 [29] and expression of the CCL20 gene was also increased 3-fold in parasitized HSEs (Table 1). IL1F9 increases IL-6 and IL-8 production by epithelial cells [30] and these HSEs secreted significantly increased amount of these cytokines in response to burrowing mites [10]. IL1F9 genes and cytokines are expressed by keratinocytes during contact hypersensitivity and in psoriasis patients [31]. It is interesting that burrowing scabies mites also induce expression of this gene since these skin diseases share some common clinical features with scabies infestation.

While we found that many GO groups exhibiting a high percentage of differential gene expression were ones involved in the secretion of cytokines associated with immune and inflammatory responses, few genes involved with the expression of the receptors for these cytokines changed expression. The exceptions were the up-regulated expression of IL-1R1, IL-1R2 and IL-13RA2 genes. Although this would seem to promote inflammation when IL-1 was secreted by HSEs, we also found that the mites up-regulate secretion of IL-1ra that would block the receptor and thus the pro-inflammatory effects of IL-1 [10].

The genes in the cytokine GO group that changed expression the most were those that code for IL-1α, IL-1β, IL-11, IL-16, IL-20, IL-23, and transforming growth factor-α (TGFα). All of these are multiple function cytokines and associated with inflammation in the skin. IL-16 is produced by keratinocytes [32] and is chemotactic stimulating the migration of CD4+ T-lymphocytes, monocytes and eosinophils [33], [34]. Gene expression for this cytokine was down-regulated and this could be responsible for the delay in inflammatory response observed in early scabies. IL-20 is highly expressed in inflamed tissue such as psoriatic skin [35], [36]. During inflammation, IL-20 regulates the proliferation and differentiation of keratinocytes [35], [37], [38]. Thus, up-regulation of IL-20 may contribute to the proliferation of keratinocytes and development of a thickened epidermis (hyperkeratosis) and the scaly and crusted skin associated with advanced scabies. IL-23 is produced in excess in psoriatic skin [39]–[44]. *In vivo*, IL-23 is produced by keratinocytes and dendritic cells and its effect in skin pathology is mediated by Th17 cytokines [44]. It has been suggested that blocking IL-17 and IL-23 may suppress chronic inflammatory diseases [41], [44]. Ustekinumab, that blocks IL-23 and IL-12, has been shown to be effective in treating psoriasis [45]. Our finding that the genes for IL-20 and IL-23 are highly expressed in response to burrowing mites raises the question – would blocking these cytokines be a possible novel treatment for reducing the inflammation and itch associated with scabies?

Our earlier studies showed that monocultures of keratinocytes and/or fibroblasts in response to scabies extracts secreted cutaneous T cell-attracting chemokine (CTACK, CCL27), thymic stromal lymphopoietin (TSLP), growth-related oncogene-α (GROα), transforming growth factor-α (TGFα), IL-1β, IL-3, IL-6, IL-8, IL-10, monocyte chemoattractant protein-1 (MCP-1, CCL2), G-CSF, GM-CSF, macrophage colony-stimulating factor (M-CSF), VEGF and thymus- and activation-regulated cytokine (TARC, CCL17) [10]. Mites burrowing into HSEs significantly up-regulated secretion of CTACK, TSLP, IL-1α, IL-1β, IL-1ra, IL-6, IL-8, MCP-1, G-CSF, GM-CSF, and M-CSF [10]. We did not attempt to measure IL-3 and IL-10 secretion by HSEs. Thus, the HSEs did not significantly up-regulate secretion of GROα, TGFα, VEGF, and TARC as did extract-challenged keratinocytes nor did mite-challenged HSEs exhibit a 2-fold change in the expression of the genes for these four cytokines. Placing soluble mite extract on the HSE surface did not significantly stimulate secretion of most of these cytokines [10] nor did it up-regulate cytokine gene expression for any cytokine other than IL-1β. The differences in the responses of HSEs and monocultured cells and between burrowing mites and mite extracts are difficult to interpret because there can be multiple contributing factors. In the instance of cultured cells, extract molecules in the culture media directly stimulate cells. The composition and concentration of modulating molecules likely varies between what is in an extract and what is released by a burrowing mite. Also, isolated cells (keratinocytes and fibroblasts) may respond differently from these same cells in HSEs that can have cell-cell interactions and interactions with the matrix. Live burrowing mites that penetrate the epidermis of HSEs provide a physical stimulus while extracts placed on the surface of HSEs or in culture media do not. There are undoubtedly differences in the local concentration of molecules from the live mite secretions (area around the mouth parts and anus) and an extract although the later likely contains all the molecules that the mite secretes. Also, the nature of the monoculture experiments are such that the cytokines that are measured have accumulated over time in the culture medium while gene expression only represents the genes expressed at the instant the tissue is harvested.

The chemokine genes whose expression was most changed were CCL3 (MIP-1α), CCL5 (RANTES), CCL20 (MIP-3α), CXCL2 (MIP-2α), CXCL5 (ENA-78), CXCL6 (GCP-2), CXCL12 (SDF-1), and CXCL14 (BRAK). All of these genes are members of the taxis GO group. The secretion of these chemokines was not determined. This gene expression is consistent with histological studies of scabietic lesions that show inflammatory cellular infiltrate that concentrates around the mouth parts and anal opening of the burrowing mite [46]. This suggests that the effector molecules responsible for this are associated with salivary secretions and fecal material and that these induce chemotactic responses of cells in the vicinity of the mites. However, CXCL12 and CXCL14 were both down-regulated by >2-fold. CXCL12 stimulates the migration of T-lymphocytes, monocytes and neutrophils [47], [48]. CXCL14 (BRAK) is a chemoattractant for macrophages, immature dendritic cells and natural killer cells [49]. Early down-regulation of these cytokines would be advantageous for the mite as it is attempting to become established in the host skin.

Another GO group with many genes affected by burrowing scabies mites was the keratinization group. This is not surprising because hyperkeratosis with scaly and crusted skin develops as scabies infestations progress over time. Ten genes associated with keratinization were up-regulated. Most of these genes (LCE2D, LCE3A, LCE3C, LCE3D) are associated with cornification of the epidermis to become stratum corneum. This is not unexpected given that the epidermis is damaged by the burrowing mite. The KRT37 gene was the most up-regulated gene expressed. The KRT37 protein is a type I keratin found in hair and nails. The significance of the change in expression of this gene is unclear but it may be involved in the repair of the epidermis that is damaged by mite burrowing.

### Summary

This study has shown that a large number of genes in skin keratinocytes and fibroblasts are differentially expressed in response to live burrowing scabies mites, their products and physical activity. The HSEs used in this study consisted of only two cell types (keratinocytes and fibroblasts). The fibroblasts and keratinocytes in these HSEs seem to be responding to the invading mites and their products with the expected pro-inflammatory response. *In vivo*, many other cell types also respond to the live scabies mites and their products as well as to the substances produced by keratinocytes and fibroblasts in response to the mites. Thus, there is a complex interaction among many cell types in the skin (including antigen-presenting cells and lymphocytes) in response to scabies. Most of these interactions remain to be identified but those already elucidated involve other cell types including endothelial cells, LCs, dendritic cells and lymphocytes. Likewise, many of the adaptations that scabies mites have evolved to modulate and exploit a host remain to be identified. However, it is clear that these mites are capable of modulating many aspects of the innate and adaptive protective responses of their host enabling them to survive and thrive.
